# Parental satisfaction with the quality of services provided to persons with autism spectrum disorder and their families in Saudi Arabia

**DOI:** 10.3389/fpsyt.2024.1469513

**Published:** 2024-12-10

**Authors:** Salwa R. Saleh, Hiam M. Alaoufi, Mahmoud Mohamed Eltantawy

**Affiliations:** ^1^ Department of Teaching and Learning, College of Education and Human Development, Princess Nourah bint Abdulrahman University, Riyadh, Saudi Arabia; ^2^ Department of Special Education, College of Education, Ain Shams University, Cairo, Egypt

**Keywords:** parents, satisfaction, quality, services, autism spectrum disorder, KSA

## Abstract

The services provided to persons with autism spectrum disorder (ASD) and their families are considered the basic factors that help families meet their needs and those of their children. These services help persons with ASD attain an appropriate level of independence within the society. Therefore, this study aimed to assess the Parents’ satisfaction with the quality of services related to family guidance and education, diagnostic and evaluation services, support and intervention services, and services that aim to improve equal opportunities and life quality among persons with ASD and their parents in Saudi Arabia. This study also aimed to identify the differences in the quality of these services with regard to the age stage of the person with ASD. The study sample consisted of 301 parents of persons with ASD, including 93 fathers and 208 mothers. In this study, a quantitative approach through a questionnaire was used to collect data. Results of this study showed that the parents participating in the study reported the availability of services at an unsatisfactory level with regard to family guidance and education services, support and intervention services, and services that help to improve life quality and equal opportunities. Although the quality of diagnostic and evaluation services was satisfactory, the results indicated no differences in the quality of these services based on the ages of the persons with ASD. The results of this study indicated the importance of developing these services and subjecting them to international quality standards.

## 1 Introduction

Autism spectrum disorder (ASD) is a neurodevelopmental disorder that is characterized by inadequate social communication and interaction. It manifests through repetitive and obsessive behaviors, as well as restricted interests and activities (1). ASD can cause persons to engage in challenging behaviors (2). ASD is considered a lifelong and incurable disorder, and its cause remains unknown (3).

Neurodevelopmental disorders, such as ASD, can cause many problems to the individual and their family. Significantly, parents of children with ASD are exposed to many psychological and social problems (4). Furthermore, ASD affects the lives of all family members (5, 6).

Parents who care for someone with ASD experience higher levels of stress than those of other people (7). They face multiple challenges in different aspects of care, leading to parental stress and poor adjustment (8). They could suffer from symptoms of depression and various health problems, as having a child with ASD places a great burden on the family (9). It influences parents’ careers, as it leads to postponing their professional goals or even abandoning them, changing their future professional plans, and hindering the self-exploration as well as discovering their abilities (10). The level of care provided to parents of persons with ASD may be associated with high levels of parental stress, anxiety, and depression (11).

The support provided to parents, including information, counseling, and therapy, can play a vital role in the lives of families of persons with ASD. However, little is known about the support experiences of parents and caregivers (12). Studies have shown that persons with ASD and their families often face barriers in accessing related services and support (13). Cultural, social, and economic factors play an important role in providing services to persons with ASD. These may include ethnic background, parental income, perceived home cultures toward ASD, social stigma, and other factors (14). The support and quality of services are related to urban and rural areas, with a worse provision in rural areas (15).

Several needs of parents of persons with ASD have been highlighted through the results of different studies. These needs may be related to diagnosis, treatment, rehabilitation, and parents themselves, such as the need for knowledge and awareness of ASD; developing life skills; overcoming daily parental problems; family and marital problems; financial, cultural, and social needs (16); and information-related needs, as families lack knowledge of how to deal with the child’s behavior and future expectations. Other needs include children’s rights, sources of obtaining information, including information about the support that families can access (17). In addition, there is a need to improve access to services for ASD, creating flexible support in an inclusive environment, addressing social stigma, and changing societal attitudes (13). Parents face many problems with their own mental health issues related to cultural norms and expectations in the society (18), as well as needs related to the availability of resources, guidance, daily management, relational and emotional support (19). These parents are in need of early intervention services (20), ASD-related genetic services that include family members (21), and behavior modification counselling (22). Most of the families of children with ASD obtain basic health and education services, but the provision remains limited, including more specialist agencies, such as employment services (23). The level of family satisfaction with the services provided to them is low, such as support groups (support for siblings and support for parents), as well as support with rest periods, as families reported that these services are important, but they are not received (24).

Moreover, parents of persons with ASD report challenges in accessing and using public health services because of lack of understanding on the part of health service providers (25), the excessive workload of specialists, the lack of professional experience, poor specialized care, and the lack of recognition of the family as a unit of care (26). The presence of difficulties in diagnosis is also closely related to parental stress, as it increases or reduces the perceived stress of parents (7). The lack of experience with ASD among service providers is reported to be another challenge faced by these families (27), as they suffer from strained relationships, and parents want resources to guide, support, and educate them throughout the journey of autism (28).

The process of obtaining positive outcomes for persons with ASD requires an integrated service system and a long-term service progression that covers the person’s entire life. Educational, specialized health care and other services must be allocated and budgeted early to provide satisfactory service outcomes to persons with ASD (27). Care provision must be centered not only on the child with ASD but also on the entire family, addressing and supporting its needs, to ensure the family’s well-being and life quality (8).

A number of researchers and specialists have focused on training parents, as the results indicated the necessity of developing support programs that focus on the needs of parents and reflect the educational, behavioral, and psychological dimensions (19). These programs help parents and increase their confidence regarding areas of supporting the needs of children with ASD (29). Training programs should adopt a multidisciplinary approach and should be shared and made available through health, educational, and legal organizations, thereby providing parents with the information, knowledge, skills, and confidence to advocate for their children with ASD. Although this approach cannot guarantee that families will receive the appropriate services and support for their children’s needs, it may help them cope with potential challenges and problems (30). Formal training is also necessary for clinicians to gain confidence and applicable skills when working with this category of patients (31).

### 1.1 Models and conceptual frameworks impacting service delivery

Various models and conceptual frameworks influence the delivery of services to individuals with ASD and their families. These models and frameworks encompass: Social-Ecological Model, Stress-Coping Model, Family Systems Theory, or Quality of Life Framework.

#### 1.1.1 Socio-Ecological Model

Describes the interconnected levels of environment or context through which individuals navigate across their lifespan. Individuals and their environments exert mutual influence on one another (32). According to this model, personal, interpersonal, organizational, community, policy levels, the physical environment, and culture all play a role in shaping service delivery (33). Additional factors affecting service provision for individuals with ASD include gender, severity of disability, insurance coverage, and family income (34). This model encompasses a range of influences, such as family-level factors, personal factors, institutional factors, community factors, and policy-level factors, all of which impact the delivery of services to individuals with ASD and their families (35).

#### 1.1.2 Stress-Coping Model

In recent decades, there has been growing attention to the role of parents in their children’s development, particularly as they are seen as the primary caregivers most involved in raising their children and managing family responsibilities. This is especially relevant for parents of children with ASD (36). The goal of the Model is to help families reduce stress, reduce the amount of time they spend caring for their children, and build healthy coping skills. The model includes several strategies tailored for parents of children with ASD, such as psychoeducation, parent training, effective coping strategies, self-care strategy, eco-map strategy, group therapy strategies, individual therapy for parents and social support strategy (37). Parents deal with stress in different ways based on the pressures they experience, which are affected by their partner’s support and their own strength. One significant aspect that affects the family’s well-being is the severity level of the disability (38).

#### 1.1.3 Family Systems Theory

This theory focuses on supporting families of individuals with intellectual disabilities or ASD. The core idea behind interventions based on Family Systems Theory is that families operate as complex, interconnected systems in which members have a reciprocal influence on one another (39, 40). The goal of systemic interventions is to enhance family system functioning and address challenges faced by individual members by targeting family interactions and the beliefs they hold (41). Cridland et al. (42) emphasized the significance of this approach for families of individuals with ASD, advocating for further research grounded in this model.

#### 1.1.4 Quality of Life Framework

The quality of life for kids, teens, and people with ASD is often much lower than for their peers who do not have ASD. Differences between studies are difficult to establish because different methods are used to judge people’s quality of life (43). The symptoms and characteristics of ASD appear to impact parents similarly across different countries, and parental quality of life is influenced by the prevailing cultural context (44). Social support is critically important and should be integrated into interventions designed to enhance family satisfaction with quality of life (45). Furthermore, factors such as the child’s level of independence, the severity of the disorder, family income, and changes in professional or family circumstances can have negative effects on parental quality of life (46).

### 1.2 The current state of service provision in Saudi Arabia

A few studies have been conducted in Saudi Arabia to recognize the challenges faced by parents of children with ASD. The government facilities that provide services to these children are acceptable; however, parents of children with ASD are in need of psychological, social, emotional, and financial services (47). In addition, parents of children with ASD in the primary stage were dissatisfied with the educational services provided in regular schools, and they felt that the methods of evaluating children with ASD and the school curricula should be modified, resource rooms should be provided, and group work should be activated (48). Moreover, workers at centers for persons with ASD explained that there are deficiencies in home care, communication, and motivational programs provided to families to participate in the programs provided to their children. The increasing number of children and the work on environmental issues represent another problem facing this category (49). Regarding speech-language services (SLS) provided to children with ASD in various health facilities, parents highlighted the high costs of sessions, the need for initial behavioral sessions, and the lack of qualified SLS specialists, improvement, and specialized centers (50).

The current study attempts to fill a gap in prior research undertaken in Saudi Arabia on services offered to people with ASD and their families. This study investigated parental satisfaction with features of services that had not been addressed in earlier studies. This includes the quality of counseling and educational services, as well as services that promote equity and quality of life. Furthermore, the study examined parental satisfaction with service quality in relation to their children’s developmental phases, which varied from early childhood to adulthood. Importantly, individuals’ needs and requirements alter throughout their lives. As a result, services accessible to young people with ASD may differ greatly in quality and character from those provided to adults. To the best of the researchers’ knowledge, no one has previously investigated this factor.

Essentially, the needs and challenges of parents of persons with ASD highlight the necessity to provide medical, educational, and counseling services, as well as emotional and material support. Moreover, planning and implementing support programs enable parents to confront problems strategically, thereby improving their life quality (16). Despite the value of this support, parents of persons with ASD emphasized the limited availability of services (51). Thus, a clear pathway for support after an ASD diagnosis, special training for professionals and service providers, and the provision of specialists specifically prepared to meet the needs of children with ASD are urgently needed (12). The parents recommended improving the quality, quantity, accessibility, and availability of services; educating and training professionals to work effectively with children with ASD; increasing funding for services; and creating appropriate school places and educational programs (52).

There is an urgent need to determine the type and quality of services provided to persons with ASD and their families and to know the aspects that need to be developed. This problem is addressed through a systematic research that considers local developments represented by the programs of the Kingdom of Saudi Arabia’s Vision 2030, specifically the human capability development program and the life quality program, as well as the best international practices in the field of services for persons with ASD. All these need to be considered in future development plans to meet the aspirations of families in the type and quality of services provided to them and their children. Thus, the current study aims to evaluate parent’s satisfaction of quality of family guidance, education services, diagnostic and evaluation services, support and intervention services, and services that promote equal opportunities and improve the life quality of parents of persons with ASD in the Kingdom of Saudi Arabia. This study also aims to identify differences in the quality of these services with regard to the age stage of persons with ASD. The results of the current study may benefit Saudi government Ministries such as the Ministry of Health, the Ministry of Human Resources and Social Development, and the Ministry of Education, in making decisions that can improve the services provided to persons with ASD and their families.

## 2 Materials and methods

### 2.1 Method

The study employed a quantitative survey approach to analyze the phenomenon in question. The objective was to evaluate the satisfaction levels of parents with children diagnosed with ASD concerning the quality of services offered to them and their families. Furthermore, it aimed to uncover possible differences in service quality related to the age of individuals with ASD. A questionnaire was utilized as the primary tool for gathering data and conducting further analysis to meet these objectives.

### 2.2 Participants

Approximately 1,349,585 individuals in the Kingdom of Saudi Arabia have impairments, constituting 5.9% of the total population. Approximately 196,611 individuals are predicted to have communication problems, including those with ASD (53). The study sample was derived from families with children receiving services from the Charitable Society of Autism Families in Riyadh, which assists roughly 4,492 families (54). The study sample consisted of 301 parents of persons with ASD, including 93 fathers, accounting for 30.9%, and 208 mothers, accounting for 69.1%.

A total of 189 parents with a son diagnosed with ASD, 105 parents with a daughter diagnosed with ASD, and 7 parents with both a son and a daughter diagnosed with ASD were included in the study. The children’s severity of ASD varied, with 217 cases classified as mild, 58 as moderate, and 26 as severe. The mean age of the children was 11.4 years, and their ages ranged from 3 to 21 years. The majority of diagnoses were obtained from licensed clinics accredited by the Saudi Ministry of Health or services provided by the Ministry.

The education level of the participants was as follows: 122 participants with secondary school education and below, accounting for 40.53%; 148 participants with a university-level education, accounting for 49.2%; and 31 participants achieved postgraduate qualifications, accounting for 10.3%. As for the distribution of participants within the regions of the city of Riyadh, the number of participants from the central region was 176 persons, accounting for 58.53%, and 21 participants were residing in the northern region, accounting for 7%. In addition, 59 participants lived in the western region, accounting for 19.6%; 22 persons inhabited the Eastern Region, accounting for 7.3%, and 23 participants inhabited the Southern Region, accounting for 7.6%. All areas are located in the city of Riyadh and are characterized by varying economic and social levels.

### 2.3 Tools

A questionnaire was prepared to identify the quality of services provided to persons with ASD from the point of view of their parents. In its final form, the questionnaire consisted of 24 items. The researchers conducted a pilot questionnaire by presenting it to 15 parents of these persons and specialists in the field of ASD to determine their understanding of the questionnaire’s vocabulary, the required modifications from their point of view, and the services included in the questionnaire and their suitability. This step resulted in deleting some items, modifying others, and adding some services. The questionnaire consisted of two main parts. The first one includes general information such as educational qualification, job, residential area, kinship with the person with ASD, age of the person with ASD, and consent to collect information and use it for research purposes. The second part consists of the questionnaire items, which are divided into four main dimensions as follow: The first dimension, family guidance and education, includes introducing growth stages and care methods. It also includes the basics of first aid, introduction to ASD and its effects on families at various ages, reference to ASD families’ legal and material rights, intervention methods, and therapeutic programs. The second dimension addresses diagnostic and evaluation services, including, regular checks on fundamental growth and health, referring the person to specialists for diagnosis and support from a multidisciplinary team diagnostic, providing a detailed report to the carer/guardian with diagnosis, evaluation outcomes, and intervention priorities. The third dimension includes: support and intervention programs that introduce ASD patients and their families to basic services. These refer to support and empowerment centers in residential communities, remote family counseling (hotline), urgent intervention programs after diagnosis, offering appropriate family training and assistance programs, teaching people with ASD in classes with their peers without disabilities, providing transitional services to help people transition from one educational stage to another and to work, and providing vocational rehabilitation and training services. The fourth dimension includes: services to attain equal opportunities and quality of life including social integration services for people with ASD, enabling them to achieve independence, productivity and social integration, as well as using entertainment, retail, and sports services more easily. Also, providing ASD-specific care homes for short periods or longer, so rest and recreation should be available to ASD families. It also refers to providing continued education and training for ASD patients at all ages and insurance packages that encompass health, education and behavioral support. The questionnaire scores ranged between 24 and 96 degrees, and they are answered by choosing from four alternatives, namely, the service is completely unsatisfactory, unsatisfactory service, satisfactory service, and the service is completely satisfactory, which are graded as 1, 2, 3, and 4, respectively.

A sample of 123 parents was randomly selected from the study population to validate the psychometric properties of the questionnaire. These participants were later excluded from the final implementation of the study’s tools to avoid any influence on the psychometric property calculations. The psychometric properties of the questionnaire were verified by calculating the internal consistency of the questionnaire items by calculating the correlation coefficient between each item and the dimension to which it belongs. Based on statistical analysis using SPSS-27, the values of the coefficient ranged between 0.857 and 0.890 for the first dimension, 0.699 and 0.893 for the second dimension, 0.633 and 0.846 for the third dimension, and 0.741 and 0.892 for the fourth dimension. All values of the correlation coefficients between the degree of each individual and the degree the totality of the dimension are significant at the (0.01) level, which indicates the internal consistency of the questionnaire. The correlation coefficient was calculated between the score of each dimension and the total score of the questionnaire. The scores 0.930, 0.876, 0.971, and 0.938 indicate that the dimensions are consistent with the questionnaire as a whole, as all correlations between the dimensions and the total score were significant at the 0.01 level. This result indicates the consistency between all dimensions of the questionnaire. The stability coefficient of the questionnaire was calculated using the Cronbach’s alpha method. The stability coefficient of the dimensions was 0.923, 0.942, 0.962, and 0.968, respectively, and the overall stability coefficient was 0.982, which is consistent with the stability of the questionnaire.

### 2.4 Data collection

This study was approved by the Ethics Committee of Princess Nourah bint Abdulrahman University in Riyadh, Saudi Arabia. The questionnaire was prepared electronically and applied via the Internet, and it took about 10 min to complete. Participants were informed of the aims and procedures of the study and provided active informed consent by selecting a specific option on the online form. Participants were informed that the data would be stored and processed anonymously, that participation was voluntary, and that withdrawal was permitted at any time. Participants initially completed the general information and then responded to the questionnaire items.

### 2.5 Statistical methods

Following data collection, appropriate statistical methods were employed to effectively address the research questions. The data were analyzed using SPSS version 27 to assess the study questions. Statistical techniques such as percentage, arithmetic mean, and standard deviation (Std.) were utilized to determine the level of parents’ satisfaction with service availability. Given the absence of conditions for parametric statistics, specifically, the homogeneity of the sample and the normality of distribution, then non-parametric statistics were applied. The Kruskal-Wallis test was conducted to identify differences in quality among the groups: children under 6 years, children aged 6 to less than 12 years, and individuals older than 12 years.

### 2.6 Procedures

This study encompassed several essential procedures: initially, a comprehensive literature review was conducted to identify amenities that could improve the quality of life for individuals with ASD and their families. Then, a questionnaire was developed to evaluate parental perspectives on service quality. A study on the psychometric properties of the questionnaire was carried out to confirm its reliability and validity. A study sample was subsequently determined, and the survey was conducted. Data were gathered and subjected to statistical analysis employing suitable techniques. Subsequently, findings and results were obtained, leading to a set of recommendations that may assist service providers in improving the quality of provision for individuals with ASD and their families.

## 3 Results

The first question was related to the level of Parents’ satisfaction with the quality of services provided to families and their children. To answer this question, arithmetic means, standard deviations, and percentages were calculated for parents’ responses to a questionnaire on the quality of services provided to persons with ASD. The cell length for each level was set at 0.75, and the level of service availability was determined as follows: The service is completely unsatisfactory 1 to 1.75, unsatisfactory service 1.76 to 2.50, satisfactory service 2.51 to 3.25, and the service is completely satisfactory 3.26 to 4.00. **Table 1** shows the results related to the first question.

**Table 1:** Arithmetic means, standard deviations, and percentages of questionnaire items related to the quality of services provided to persons with ASD.

N	Item	Responses						
		The service is completely unsatisfactory %	Unsatisfactory service %	Satisfactory service %	The service is completely satisfactory %	Means	STD	Service satisfaction level
The first dimension: family guidance and education services								
1	Introducing the stages of growth and ways to care for them	26.9	18.9	35.2	18.9	2.46	1.081	unsatisfactory
2	Introduction to first aid methods	40.9	21.9	25.9	11.3	2.08	1.057	unsatisfactory
3	Introduction to ASD and its impact on persons and their families at different ages	23.9	23.6	33.2	19.3	2.48	1.057	unsatisfactory
4	Introducing the legal and material rights of persons with ASD and their families	42.2	23.6	21.6	12.6	2.05	1.070	unsatisfactory
5	Introducing various intervention methods and therapeutic programs	21.6	24.9	31.2	22.3	2.54	1.063	satisfactory
The second dimension: diagnostic and evaluation services								
6	Periodic follow-up of the basic stages of growth and verification of the absence of health problems	24.6	25.9	27.2	22.3	2.47	1.091	unsatisfactory
7	Referring the person to specialists to identify problems and provide support	19.9	28.6	29.2	22.3	2.54	1.047	satisfactory
8	Diagnosis by a multidisciplinary team	19.3	24.9	32.9	22.9	2.59	1.043	satisfactory
9	Providing the guardian with a comprehensive report that includes the diagnosis, evaluation results, and intervention priorities	18.3	24.9	31.2	25.6	2.64	1.054	satisfactory
The third dimension: support and intervention services								
10	Providing an introductory guide to the basic services provided to persons with ASD and their families	31.6	22.6	25.9	19.9	2.59	1.043	satisfactory
11	Providing support and empowerment centers in residential neighborhoods	52.2	20.9	14.6	12.3	2.64	1.054	satisfactory
12	Providing remote counseling services for families (hotline)	37.9	23.3	21.9	16.9	2.34	1.122	unsatisfactory
13	Providing intervention programs immediately after obtaining the diagnosis	33.2	23.6	24.6	18.6	1.87	1.071	unsatisfactory
14	Providing various family training and guidance programs that suit the person’s age and family needs	33.2	24.6	27.9	14.3	2.18	1.117	unsatisfactory
15	Teaching persons with ASD in classes with their peers without disabilities	29.2	29.9	24.3	16.6	2.29	1.116	unsatisfactory
16	Providing transitional services that contribute to the smooth transition of a person from one educational stage to another and the transition to work	41.2	21.3	23.3	14.3	2.23	1.064	unsatisfactory
17	Providing vocational rehabilitation and training services	35.2	25.2	24.6	15.0	2.28	1.060	unsatisfactory
The fourth dimension: services to achieve equal opportunities and quality of life								
18	Providing social integration services for persons with ASD	31.9	31.9	18.9	17.3	2.11	1.099	unsatisfactory
19	Employing persons with ASD to achieve independence, productivity, and integration into society	38.9	27.2	18.6	15.3	2.19	1.078	unsatisfactory
20	Easily access entertainment, shopping and sports services	41.2	26.6	19.9	12.3	2.22	1.075	unsatisfactory
21	Providing specialized care homes to receive persons with ASD for short periods or days as needed	52.2	21.6	14.3	12.0	2.10	1.086	unsatisfactory
22	Families with ASD have access to opportunities for rest and recreation	56.1	18.6	11.3	14.0	2.03	1.051	unsatisfactory
23	Providing continuing education and training services at all age levels for persons with ASD	37.5	26.6	19.9	15.9	1.86	1.062	unsatisfactory
24	Providing insurance that includes packages covering basic services (health, education, behavioral, and support)	49.5	21.9	14.0	14.6	1.83	1.099	unsatisfactory

**Table 1** shows the percentages, arithmetic means, and standard deviations of parents’ responses. The responses ranged from an unsatisfactory level to a satisfactory level of service.

The second question was related to the presence of differences in the quality of service provided among the three groups (the group of children less than 6 years, the group of children from 6 years to less than 12 years, and the group of persons older than 12 years). To answer this question, non-parametric statistics were used because no conditions are provided for parametric statistics, the most prominent of which are the homogeneity of the sample members and the moderation of the distribution. The Kruskal–Wallis test was used for independent samples. **Table 2** shows the results related to the second question.

**Table 2:** Results of the Kruskal–Wallis test and its level of significance for the differences between the averages of the groups’ scores in accordance with the age stage of the person with ASD.

The dimension	Age stage of a person with ASD	N	Average Ranks	Kruskal–Wallis value	Sig
Family guidance and education services	children less than 6 years	77	159.54	1.551	0.46
	children from 6 years to less than 12 years	139	144.88		
	persons older than 12 years	85	152.90		
Diagnostic and evaluation services	children less than 6 years	77	170.52	5.365	0.068
	children from 6 years to less than 12 years	139	145.72		
	persons older than 12 years	85	141.95		
Support and intervention services	children less than 6 years	77	160.99	1.418	0.492
	children from 6 years to less than 12 years	139	148.55		
	persons older than 12 years	85	145.95		
Services to achieve equal opportunities and quality of life	children less than 6 years	77	162.61	1.881	0.39
	children from 6 years to less than 12 years	139	146.31		
	persons older than 12 years	85	148.15		
Total score	children less than 6 years	77	164.70	2.457	0.293
	children from 6 years to less than 12 years	139	146.57		
	persons older than 12 years	85	146.11		

As shown in **Table 2**, no statistically significant differences in the average ranks of the groups’ scores on the questionnaire related to the quality of services provided to persons with ASD because of the various age stages of the persons with ASD.

## 4 Discussion

In this study, the parental satisfaction with the degree of availability of services provided for persons with ASD was examined, and differences in the level of their quality were identified based on the age stage of the person with ASD. This study aimed to identify the level of quality of services provided to persons with ASD in the Kingdom of Saudi Arabia in light of the researchers’ knowledge. Previous studies conducted within the Kingdom of Saudi Arabia have focused on the evaluation of a special group of services that differ from those examined in the current study, which addressed services from an integrative perspective of the parents’ needs, particularly services that enable them to care for their children. Helkkula et al. (27) indicated that the process of obtaining positive outcomes for persons with ASD requires the existence of an integrated service system and a long-term service process. Furthermore, the scope of the service has to cover the person’s entire life.

With the expansion and development of educational and behavioral theory, shifts in how and where services are provided to persons with ASD are observed, whether at school or after school, as well as in the methods and practices that are used to reach those results. These transformations require new knowledge and accelerating appropriate interventions (55).

The results indicated that the participants, both mothers and fathers, reported the availability of services at levels ranging from unsatisfactory to satisfactory. The questionnaire included 24 services, of which 18 obtained an unsatisfactory level, whereas only 6 obtained a satisfactory level. This finding indicates that these services need considerable development and attention to meet the needs of parents, and that the current level of services is below the required level. Attention should also be paid to the participation of parents and supporting them through family guidance and counseling programs, training programs, and support groups. As stated by Hayes and Watson (6), ASD affects the lives of all family members.

The results indicated highly significant deficiencies in family guidance and education services. Remarkably, a high number of provisions for families caring for a person with ASD were found unsatisfactory. These included services for introducing the stages of development and ways to care for them, teaching first aid methods, introducing ASD and its impact on persons and their families at different age levels, and introducing the legal and material rights of persons with ASD and their families. However, the service of introducing various intervention methods and therapeutic programs achieved a satisfactory level of service. This result indicates the low quality of these services, despite of their importance to parents in helping them to adjust to life of caring for someone with ASD. Weissheimer et al. (17) indicated the need for information for families with ASD, as families lack knowledge of how to manage the child’s behavior, protect their rights, find sources of obtaining information and support. Khan et al. (47) indicated that public awareness of ASD is very poor. Murphy and Tierney (30) also pointed out the importance of providing these services through a multidisciplinary approach. Diagnostic and evaluation services are considered good services that have met parents’ satisfaction with regard to the level of service, as most of their services reached a satisfactory level. The service of referring the person to specialists to identify problems and provide support, the diagnosis service by a multidisciplinary team, the service of providing the parents or a guardian with a comprehensive report that includes the diagnosis and evaluation results, and the intervention priorities were at a satisfactory level. However, the periodic follow-up service for the basic developmental stages and verifying the absence of health problems were at an unsatisfactory level of service. In general, the diagnostic and evaluation services were at a satisfactory level. This result is consistent with the result of Khan et al. (47) conducted in the Kingdom of Saudi Arabia, which indicated that children were diagnosed and provided with services early. Therefore, there is an interest in the process of diagnosing persons with ASD. In the same context, Shivers et al. (23) indicated that families often obtain basic services such as health and education, but a number of services remain limited and may not be provided to families and their children.

With regard to support and intervention services, they received an unsatisfactory general level. These included: the service of providing remote consultations for families (hotline); providing intervention programs immediately upon obtaining a diagnosis; providing various family training and guidance programs in proportion to the age stages of the person, as well as the needs of the family; educating persons with ASD in classes with their non-disabled peers while providing them with appropriate support; providing transitional services that contribute to the smooth transition of the person from one educational stage to another and the transition to work; and providing rehabilitation and vocational training services showed an unsatisfactory level. By contrast, the service of providing an introductory guide to the basic services offered to persons with ASD and their families and enabling support and empowerment at centers in residential neighborhoods achieved a satisfactory level. This result can be explained by the fact that basic services are available in a satisfactory manner, whereas specialized services are not because of the lack of specialists or the lack of training to meet the needs of persons with ASD. According to Khan et al. (47), government facilities providing services to persons with ASD in the Kingdom of Saudi Arabia are acceptable; however, parents of children with ASD are in need of many other services.

As for the services of equal opportunities and achieving life quality, they were all found unsatisfactory to parents. The services of providing social integration to persons with ASD; employing persons with ASD to achieve independence, productivity, and integration into the society; enabling easy access to entertainment, shopping, and sports services; providing specialized care homes to persons with ASD for short periods or longer, as needed, in order to provide rest and recreation opportunities to families with ASD; providing continuous education and training at all age levels for persons with ASD; and providing an insurance service that includes packages covering basic services (health, educational, behavioral, and support), all recorded unsatisfactory levels. This result indicates an interest in basic services without giving importance to other services related to the life quality of families and the reduction of the burden placed on them as a result of the disorder and the restrictions it imposes.

In general, the result of the current study is consistent with that of many studies conducted in different countries, such as the results of the study by Zunaa et al. (24), which showed that families had a low level of satisfaction with the services provided to them, Milosevic et al. (12), noted that support for persons with ASD is widely disparate and not equitably accessed, and Damiao et al. (51), similarly pointed out the limited availability of service providers.

As for the presence of differences in the parents’ responses to the questionnaire in relation to the different age stages, the results showed no differences caused by the age stage. This result can be explained by the fact that the services provided to parents with ASD are generally unsatisfactory. Notably, providing high-quality specialized services requires qualified professionals and strong and sustainable government support. These services include social integration services, employment, recreation, shopping and entertainment, family entertainment and leisure services, general and continuing education, including professional training.

The social-ecological model states that a variety of elements influence service delivery, including personal interactions, organizational, societal and political dimensions, as well as physical and cultural environments (33). Other factors related to gender, severity of disability, family income and insurance coverage (34), factors related to the family’s socio-economic level, institutional factors and community factors (35). Importantly, the launch of Saudi Vision 2030 and its programs and indicators aimed at improving the quality of life of individuals and the services provided to them in general has raised family expectations regarding the quality and standards of services more than before. According to the quality-of-life framework, social support plays a crucial role and should be considered in interventions aimed at improving satisfaction with the quality of family life (45). Saudi Arabia has clear government plans to overcome such obstacles to the quality of service in general and the services provided to individuals with disabilities in particular. A good example of these efforts is the establishment of the General Entertainment Authority, one of whose objectives is to provide entertainment services to individuals with disabilities and their families.

Notably, the quality and nature of services vary in accordance with the age stage of the child and the family. Therefore, with regard to family counseling services, equal opportunities, support, and intervention, parents’ satisfaction with these services is low. These services are renewable depending on the age stage, their developmental characteristics, and the way the disorder affects the individual, which requires the presence of qualified specialists who can meet the requirements of the individual and the family. For example, specialists in early intervention services cannot provide vocational guidance services to individuals aged 14 and over with regard to equality and specialization.

The study results also highlight the importance of formal training to gain confidence and applicable skills when working with this group (31 as well as the importance of providing training programs to parents. Furthermore, support groups could help reduce the social isolation of parents, provide access to relevant information, promote meaningful interactions with other parents, and allow them to learn advocacy skills and gain confidence in their roles as parents (29, 56, 57).

It is important to emphasize that cultural and social factors significantly influence the utilization of services provided. The study sample included participants from various educational backgrounds (high school or below, bachelor’s degree, and postgraduate), as well as participants from different areas of Riyadh, who vary in terms of awareness, as well as cultural, social, and economic status. Furthermore, depending on the child’s gender, parents may have varying degrees of satisfaction with the services provided. Notably, cultural and societal variables significantly influence the services offered to girls and women with ASD, particularly during childhood and early adulthood.

Saudi government entities such as the Ministry of Health, the Ministry of Human Resources and Social Development, and the Ministry of Education, can benefit from study finding in establishing quality services. For example, they could develop e-learning platforms to deliver guidance and educational programs that parents can use easily from home. Parents could also share their stories and give each other advice by setting up an online support group that is supervised by a professional. That would make parents feel better and help them in real ways. The study also suggests that professionals providing family counselling and education services should be required to have professional licenses. These licenses should be updated every two years to make sure that professionals stay up to date on the latest changes in the field. Indicators and standards should be implemented to monitor the quality of services at the national level. In addition, they could make available more of free or low-cost services, establish partnerships with the private sector, to promote charitable organizations, especially in areas such as intervention, vocational training, and diagnosis. Lastly, it is suggested that a special fund be set up to help families pay for care and intervention. The money for the fund could come from collaboration between the government and the business sector.

Notably, a set of limitations should be considered when generalizing the results of this study. For example, this study was conducted in the city of Riyadh, which is the capital of the Kingdom of Saudi Arabia, where services are available. Moreover, the results are related to the services that the study addressed, and participants’ satisfaction may differ from other services that were not addressed in the current study, as well as social and cultural contexts, which may have a direct impact on the generalization of the study results. Therefore, future studies should include a wide range of research in diverse environmental and cultural contexts.

## 5 Conclusion and recommendations

In this study, the level of services provided to persons with ASD and their families was elucidated from the point of view of their parents, as they are primarily concerned with determining the quality of these services and who is responsible for caring for these people. Moreover, a questionnaire focusing on the services that are important to parents was presented, and the results highlighted the importance of paying attention to preparing specialists working with these persons. Another finding highlighted the necessity of continuous training during service, and the importance of providing training and guidance programs to the families of these persons to develop their awareness and hone their abilities in order to carry out their roles.

The results of the study demonstrated the importance of developing these services and subjecting them to international quality standards in the field of ASD, as well as the importance of conducting further studies related to the level of availability of these services from different points of view. For example, focusing on caregivers themselves and persons with ASD, addressing other services that were not addressed in the current study, and conducting studies in other areas of the Kingdom other than the city of Riyadh are all relevant directions for further research. From the methodological perspective, using approaches other than the descriptive approach, such as the qualitative approach and the mixed method should be considered Moreover, conducting more studies related to the services provided to women is essential especially during adolescence and early adulthood, because there is a scarcity of studies dedicated to women at these age stages, as cultural and social factors play a major role in providing services to this disadvantaged group.

## Data Availability

The raw data supporting the conclusions of this article will be made available by the authors, without undue reservation.
